# Low Preoperative Lymphocyte-to-Monocyte Ratio Is Predictive of the 5-Year Recurrence of Bladder Tumor after Transurethral Resection

**DOI:** 10.3390/jpm11100947

**Published:** 2021-09-23

**Authors:** Kyungmi Kim, Jihion Yu, Jun-Young Park, Sungwoon Baek, Jai-Hyun Hwang, Woo-Jong Choi, Young-Kug Kim

**Affiliations:** Department of Anesthesiology and Pain Medicine, Asan Medical Center, University of Ulsan College of Medicine, Seoul 05505, Korea; kyungmi_kim@amc.seoul.kr (K.K.); yujihion@gmail.com (J.Y.); parkjy@amc.seoul.kr (J.-Y.P.); baekhans@naver.com (S.B.); jaehyun.hwang.uucm@gmail.com (J.-H.H.)

**Keywords:** bladder tumor, lymphocyte-to-monocyte ratio, peripheral blood parameters, tumor recurrence, transurethral resection

## Abstract

Many studies have investigated the prognostic significance of peripheral blood parameters—including lymphocyte-to-monocyte ratio (LMR)—in several cancers in recent decades. We evaluated the prognostic factors for five-year tumor recurrence after the transurethral resection of a bladder tumor (TURBT). In total, 151 patients with non-muscle invasive bladder tumors who underwent TURBT under spinal anesthesia were selected for this retrospective analysis. The time to tumor recurrence was determined by the number of days from surgery until there was a pathological confirmation of tumor recurrence. The preoperative and postoperative laboratory values were defined as results within one month prior to and one month after TURBT. Univariate and multivariate Cox regression analyses were performed. Seventy-one patients (47.0%) developed recurrent bladder tumors within five years after the first TURBT surgery. The multivariate Cox regression analysis revealed that preoperative LMR (hazard ratio, 0.839; 95% confidence interval, 0.739–0.952; *p* = 0.006) and multiple tumor sites (hazard ratio, 2.072; 95% confidence interval, 1.243–3.453; *p* = 0.005) were independent recurrence predictors in patients with recurrent bladder tumors within five years after the TURBT. A low preoperative LMR is an important predictor for the recurrence of a bladder tumor during a five-year follow-up period after surgery.

## 1. Introduction

A bladder tumor is among the top 10 most common tumors for both genders; in 2018, there were 549,000 newly diagnosed cases and 200,000 deaths worldwide [1]. In particular, a bladder tumor is associated with the ninth highest mortality rate in men. Almost three-quarters of all bladder tumors are non-muscle invasive bladder cancers; this tumor is also well-known for its wide range of tumor biology and heterogeneity. These heterogeneities of non-muscle invasive bladder tumors contribute to a high recurrence rate and expensive economic burden for patients [2]. Thus, it is important to evaluate prognostic factors and prevent bladder tumor recurrence.

Many studies have investigated the prognostic significance of peripheral blood parameters in several tumors in recent decades. They revealed that the inflammatory response is a determining factor of tumor progression and recurrence [2]. While tumors maintain the progress of disease and promote carcinogenesis, a systemic inflammatory response is an essential process and accomplishes the full malignant phenotype, such as tumor tissue remodeling, angiogenesis, metastasis, and the suppression of the innate anticancer immune response [3]. The lymphocyte-to-monocyte ratio (LMR), neutrophil-to-lymphocyte ratio (NLR), platelet-to-lymphocyte ratio (PLR), and red cell distribution width are valuable prognostic markers for solid tumors [4,5]. In particular, a low LMR is associated with a high tumor mutational burden and an insufficient immune reaction [6,7]. Furthermore, a low LMR is associated with a poor prognosis in several cancers [8,9]. However, no studies to date have reported the association between the LMR and tumor recurrence rate after transurethral resection of bladder tumor (TURBT).

Our previous study found that the five-year recurrence rate was lower in patients who underwent spinal anesthesia for non-muscle invasive bladder tumor resection than in those who underwent general anesthesia [10]. Thus, we designed this study to include only patients who underwent TURBT under spinal anesthesia, and evaluated independent prognostic factors including the peripheral blood parameters for the five-year recurrence of bladder tumor after TURBT.

## 2. Materials and Methods

### 2.1. Patient Characteristics

**Figure 1:**
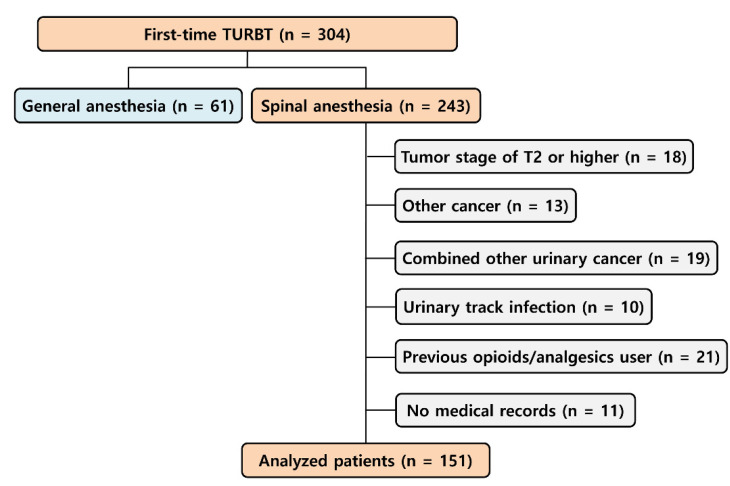
Flowchart of study patients. In January 2000–December 2007, 304 patients who underwent first-time elective TURBT at our institution were assessed. Finally, 151 patients were subjected to the study protocol. TURBT, transurethral resection of bladder tumor.

In total, 304 patients who underwent elective TURBT for the first time for non-muscle invasive bladder tumors at Asan Medical Center in Seoul, Korea were selected in January 2000–December 2007. However, 153 patients who underwent general anesthesia, had a tumor stage of T2 or higher, had suffered from other cancer, had combined other urinary cancer, had suffered a urinary tract infection, had taken opioids or analgesics before surgery, or had no medical records within the five-year postoperative period were excluded from the statistical analysis. In total, data from 151 patients who underwent TURBT for the first time were analyzed (Figure 1). The Asan Medical Center Institutional Review Board waived written informed consent and approved this retrospective study (approval number of 2017-1155). This study was performed in accordance with the Strengthening the Reporting of Observational Studies in Epidemiology (STROBE) criteria [11].

### 2.2. Spinal Anesthesia

After essential basic monitoring (electrocardiography, noninvasive blood pressure, and pulse oximetry), all patients underwent spinal anesthesia using 0.5% heavy bupivacaine (8–10 mg). Neuraxial blockade was confirmed by the loss of temperature or pin prick sensation at 10 min after spinal anesthesia. If a patient requested sedation, midazolam (2–5 mg) was administrated intravenously.

### 2.3. Clinical Data Collection

Tumor recurrence was defined by the pathological confirmation of a newly developed tumor. The time to tumor recurrence was determined by the number of days from surgery until the confirmation of tumor recurrence. 

Data regarding demographics, pathologic findings to confirm the tumor grade and histological variant, imaging studies to confirm the tumor stage, intervention methods other than TURBT, and perioperative laboratory values were collected.

The demographic data included age, sex, body mass index, the American Society of Anesthesiologists physical status classification, comorbidities, and smoking status. Data regarding multiple tumor sites, tumor grade, histological variant, tumor stage, chemotherapy, and Bacillus Calmette-Guérin therapy were also collected. The tumor grade was assessed by the 2016 World Health Organization grading system [12]. The tumor stage was distinguished as either multiple bladder tumors (more than two sites) or a pathologic tumor stage. Intervention methods included chemotherapy and a Bacillus Calmette-Guérin intravesical injection.

The perioperative laboratory values included the hemoglobin level, red cell distribution width, platelet count, absolute white blood cell count, differential white blood cell count (neutrophils, lymphocytes, and monocytes), and the calculated NLR, PLR, and LMR. The preoperative laboratory values were defined as the results obtained within one month prior to TURBT. The postoperative values were defined as laboratory results obtained within one month after surgery. 

### 2.4. Statistical Analysis

The continuous values are presented as the mean ± standard deviation, and categorical data are expressed as numbers (percentages). The student’s *t*-test or the Mann–Whitney U test was used to analyze continuous variables, and the Chi-square test or Fisher’s exact test was conducted to analyze categorical variables. We performed univariate Cox regression analysis and multivariate Cox regression analysis to determine the risk factors for bladder tumor recurrence. The parameters with a *p*-value < 0.05 in the univariate Cox regression analysis were included in the multivariate Cox regression analysis. In other analyses, a *p*-value < 0.05 was considered statistically significant. IBM SPSS Statistics 21.0 software (IBM, Armonk, NY, USA) was used for data management and statistical analysis.

## 3. Results

In total, 151 patients were enrolled in this study and 71 patients (47.0%) developed recurrent bladder cancer within five years after their first TURBT surgery (Figure 1).

Table 1 describes the patients’ demographic characteristics. There were significantly more patients with multiple tumor sites and Bacillus Calmette-Guérin therapy in the recurrent group than in the non-recurrent group.

Table 2 shows the preoperative and postoperative peripheral laboratory values within one month prior to and one month after TURBT. The preoperative LMR and postoperative hemoglobin differed significantly between the recurrent and non-recurrent groups.

**Figure 2:**
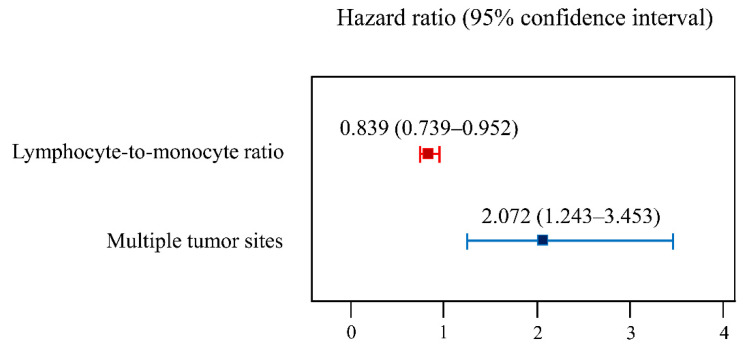
A forest plot of the multivariate Cox regression analysis to evaluate the predictors of the five-year recurrence of non-muscle invasive bladder tumor.

Table 3 presents the univariate Cox regression analysis. This analysis showed that multiple tumor sites, Bacillus Calmette–Guérin therapy, the preoperative hemoglobin and LMR, and the postoperative hemoglobin and LMR were associated with five-year recurrence after TURBT. The multivariate Cox regression analysis showed that the preoperative LMR (hazard ratio 0.839, 95% confidence interval 0.739–0.952, *p* = 0.006) and multiple tumor sites (hazard ratio 2.072, 95% confidence interval 1.243–3.453; *p* = 0.005) were independent predictors of recurrence within five years after TURBT in patients with bladder tumors (Figure 2).

## 4. Discussion

Our data revealed that preoperative LMR and multiple tumor sites are valuable for predicting five-year tumor recurrence after TURBT. This is the first study to evaluate the association between preoperative LMR and bladder tumor recurrence in patients who underwent TURBT.

Several studies have reported that peripheral inflammatory parameters, particularly the NLR, are associated with a poor prognosis of bladder cancer in patients undergoing radical cystectomy [13,14,15]. Current theories suggest that the systemic inflammatory response is an important part of carcinogenesis, in which systemic circulating immune molecules and innate immune cells are recruited, leading to the differentiation of leukocytes and causing relative neutrophilia, monocytosis, and lymphocytopenia [16]. This process might be associated with tumor angiogenesis and progression [17]. However, our study showed that the preoperative LMR was more significant for predicting five-year recurrence than the NLR. The lymphocytes in tumor progression play essential roles in anti-tumor reactions by inducing the apoptosis of tumor cells [18,19]. This finding might be due to the differences in the study populations, because our study enrolled only patients who underwent spinal anesthesia.

Hoffmann et al. suggested that lymphocytopenia is related to an inappropriate immunologic response to tumor growth [6]. Moreover, monocytes were identified as regulators in tumor growth and progression. In the early stage of tumor development, monocytes are recruited from the bloodstream to the tumor and differentiate into macrophages. These monocytes enhance tumor proliferation and tumor angiogenesis as immune suppressors and promotors of tumor neovascularization [7]. Therefore, a low LMR represents high tumor mutational burden and an insufficient immune reaction [6,7]. In accordance with our results, Hutterer et al. reported that a low LMR was potentially associated with a poor prognosis in nonmetastatic renal cell carcinoma [8]. Stotz et al. also showed that an elevated LMR was correlated with a longer time to recurrence and a higher overall survival in patients with stage III colon cancer [9].

In addition, the multiplicity of tumors was a significant, poor prognostic factor in our study. Our analysis coincides with previous studies; it showed that the risk of tumor recurrence in patients with multiple tumor sites was 4.8 times higher than those with a single tumor. Numerous studies have reported that tumor multiplicity in a non-muscle invasive bladder tumor was an independent risk factor of tumor recurrence [20,21,22,23]. According to the field effect in the tumors, the multiplicity of bladder tumors implied that the overall mucosa had the potential for malignant change [24,25]. Therefore, the multiplicity of tumors was its own factor of pathogenesis in non-muscle invasive bladder tumors and a significant, poor prognostic factor associated with tumor recurrence.

Our study has several limitations. This single-center retrospective study enrolled a small number of patients who underwent only spinal anesthesia. A previous study reported that the type of anesthetic technique affects the tumor recurrence in patients with non-muscle invasive bladder tumors [10]. In particular, spinal anesthesia is associated with a lower recurrence rate of bladder tumors than general anesthesia. Therefore, the recurrence rate among our study patients was limited. However, the results of our study may provide information regarding more influential risk factors, since we validated these risk factors in a low recurrence rate condition.

## 5. Conclusions

This study found that preoperative LMR is an independent recurrence predictor within five years after TURBT. This result suggests that preoperative LMR provides useful information on bladder tumor recurrence.

## Figures and Tables

**Table 1:** Demographic data.

	Non-Recurrent Group (*n* = 80)	Recurrent Group (*n* = 71)	*p*-Value
Age (years)	64.1 ± 13.4	63.1 ± 13.0	0.633
Male	71 (88.8)	56 (78.9)	0.120
Body mass index (kg/m^2^)	24.0 ± 3.2	23.5 ± 3.0	0.270
ASA physical status			0.191
I or II	77 (96.3)	64 (90.1)	
III	3 (3.8)	7 (9.9)	
Hypertension	21 (26.3)	21 (29.6)	0.717
Diabetes mellitus	8 (10.0)	8 (11.3)	>0.999
Smoking	28 (35.0)	27 (38.0)	0.737
Multiple tumor sites	39 (48.8)	49 (69.0)	0.014
Tumor grade			0.633
I	9 (11.4)	10 (14.1)	
II or III	70 (88.6)	61 (85.9)	
Histological variant			>0.999
Transitional cell carcinoma	79 (98.8)	71 (100.0)	
Adenocarcinoma	1 (1.3)	0 (0.0)	
Tumor stage			0.339
Ta	48 (60.0)	39 (54.9)	
T1	28 (35.0)	31 (43.7)	
Tis	4 (5.0)	1 (1.4)	
Chemotherapy	17 (21.3)	22 (31.0)	0.195
Bacillus Calmette-Guérin therapy	29 (36.3)	38 (53.5)	0.049

Data are presented as the mean ± standard deviation or number (percentage). ASA, American Society of Anesthesiologists; Tis, carcinoma in situ.

**Table 2:** Preoperative and postoperative peripheral blood parameters.

	Non-Recurrent Group (*n* = 80)	Recurrent Group (*n* = 71)	*p*-Value
Preoperative Values			
Hemoglobin (g/dL)	13.8 ± 2.2	13.2 ± 1.8	0.074
Red cell distribution width (%)	13.2 ± 1.1	13.0 ± 0.9	0.242
Platelet count (10^3^/μL)	242.4 ± 87.7	247.1 ± 125.8	0.788
White blood cell count (/mm^3^)	6707.5 ± 1961.2	6980.3 ± 2201.3	0.422
Neutrophils (/mm^3^)	4061.8 ± 1792.1	4431.1 ± 1888.0	0.220
Lymphocytes (/mm^3^)	1964.6 ± 616.8	1834.8 ± 613.0	0.198
Monocytes (/mm^3^)	432.1 ± 170.1	476.5 ± 255.6	0.206
NLR	2.3 ± 1.7	2.7 ± 1.8	0.137
PLR	134.5 ± 65.1	141.9 ± 77.2	0.522
LMR	5.1 ± 2.2	4.4 ± 1.8	0.030
**Postoperative Values**			
Hemoglobin (g/dL)	12.7 ± 2.1	12.1 ± 1.9	0.046
Red cell distribution width (%)	13.1 ± 1.2	13.0 ± 1.0	0.394
Platelet count (10^3^/μL)	224.1 ± 69.5	221.5 ± 79.6	0.825
White blood cell count (/mm^3^)	7901.3 ± 2438.6	8176.1 ± 3188.9	0.552
Neutrophils (/mm^3^)	5149.9 ± 2616.8	5667.4 ± 3134.6	0.271
Lymphocytes (/mm^3^)	1920.3 ± 849.4	1706.3 ± 707.7	0.097
Monocytes (/mm^3^)	481.4 ± 173.3	538.6 ± 269.0	0.119
NLR	3.5 ± 2.8	4.1 ± 3.3	0.216
PLR	133.5 ± 70.6	150.9 ± 107.8	0.239
LMR	4.4 ± 2.4	3.8 ± 1.9	0.066

Data are presented as mean ± standard deviation. NLR, neutrophil-to-lymphocyte ratio; PLR, platelet-to-lymphocyte ratio; LMR, lymphocyte-to-monocyte ratio.

**Table 3:** Univariate Cox regression analysis for five-year bladder tumor recurrence.

Variables	Univariate Analysis	
	Hazard Ratio (95% CI)	*p*-Value
Age	1.001 (0.983–1.019)	0.934
Body mass index	0.956 (0.887–1.030)	0.956
ASA physical status		
I or II	1.0	
III	1.888 (0.864–4.126)	0.111
Smoking	1.104 (0.684–1.783)	0.686
Multiple tumor sites	2.200 (1.328–3.645)	0.002
Tumor grade		
I	1.0	
II or III	1.104 (0.566–2.157)	0.771
Tumor stage		
Ta	1.0	
T1	1.488 (0.927–2.387)	0.099
Tis	0.419 (0.058–3.048)	0.390
Chemotherapy	1.382 (0.834–2.287)	0.209
Bacillus Calmette-Guérin therapy	1.726 (1.082–2.753)	0.022
**Preoperative Values**		
Hemoglobin	0.880 (0.789–0.980)	0.020
While blood cell count	1.027 (0.921–1.145)	0.630
NLR	1.069 (0.967–1.181)	0.191
PLR	1.001 (0.998–1.004)	0.403
LMR	0.847 (0.746–0.961)	0.010
**Postoperative Values**		
Hemoglobin	0.876 (0.784–0.979)	0.019
While blood cell count	1.037 (0.956–1.124)	0.385
NLR	1.056 (0.987–1.130)	0.117
PLR	1.001 (0.999–1.003)	0.190
LMR	0.869 (0.769–0.983)	0.025

ASA, American Society of Anesthesiologists; Tis, carcinoma in situ; NLR, neutrophil-to-lymphocyte ratio; PLR, platelet-to-lymphocyte ratio; LMR, lymphocyte-to-monocyte ratio; CI, confidence interval.

## Data Availability

The data used in the present study are available from the corresponding author upon reasonable request.
